# A Rare Cause of Chronic Life Threatening Bleeding in a Girl: The Ulcerated Blind Loops

**Published:** 2016-04-24

**Authors:** Abhijeet D Sawant, Rasik Shah, Nitin Shah, Tarun Gupta

**Affiliations:** 1 Department of Paediatric Surgery, P.D. Hinduja National Hospital and MRC, Mahim, Mumbai; 2 Department of Paediatrics, P.D. Hinduja National Hospital and MRC, Mahim, Mumbai; 3 Department of Gastroenterology, P.D. Hinduja National Hospital and MRC, Mahim, Mumbai

**Keywords:** Blind loop, Small bowel atresia, Melena, Anastomotic ulcer

## Abstract

Ulceration in a blind loop can lead to massive gastrointestinal tract (GIT) bleeding. A 13-year old girl presented with symptomatic melena requiring repeated blood transfusion since childhood. She was an operated case of small bowel atresia in neonatal life. Her upper and lower gastrointestinal endoscopies were normal. Operation showed presence of multiple ulcers in two blind loops (parts of previous side to side anastomosis) and at the anastomotic site. She underwent resection and end-to-end anastomosis of the small bowel leading to complete resolution of melena and anemia.

## CASE REPORT

A 13-year old girl presented with history of intermittent melena and bleeding per rectum requiring multiple blood transfusions since the age of 5 year. She was an operated case of small bowel atresia at day-2 of life. She was vitally stable but markedly pale. There were no significant findings on clinical examination. Her Meckel’s and RBC tagged scans were normal. Her upper and lower gastrointestinal flexible endoscopies were reported as normal. A CT scan of abdomen and pelvis showed presence of solitary dilated small bowel loop with air-fluid level near the central abdomen. At the time of presentation her hemoglobin was 2.7 gm %. She was transfused with three units of blood. After optimization she underwent operation. At exploration after extensive adhesiolysis two blind loops close to the side-to-side anastomosis found in the proximal ileum (Fig. 1). Enteroscopy performed through the blind loops revealed presence of a large circumferential anastomotic ulcer and multiple ulcers in both blind loops. The rest of the small bowel was normal. Resection of both blind loops and side-to-side anastomosis was done. Following resection end-to-end anastomosis of proximal and distal small bowel was performed. Postoperative course remained uneventful. Histopathology confirmed the presence of anastomotic ulcer with severe submucosal inflammation and reactive lymphoid hyperplasia. At one year follow up; she is asymptomatic with no history of melena and bleeding per rectum with hemoglobin level of 13 gm/dl without any need of blood transfusion.

**Figure:**
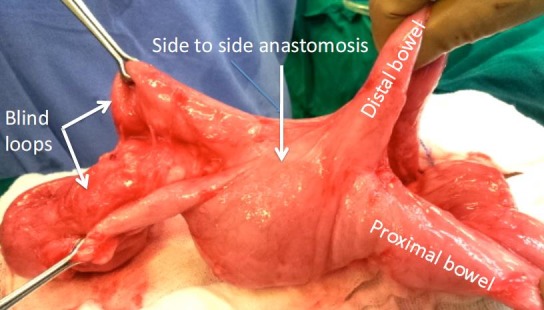
Figure 1:Figure shows two blind loops close to side to side small bowel anastomosis.

## DISCUSSION

Melena and bleeding per rectum is often a challenging condition as to the diagnosis and treatment. These patients frequently need extensive evaluation by imaging studies to establish diagnosis for its effective treatment. Flexible gastrointestinal endoscopy, technetium nuclear scan, GIT contrast studies, CT scans, CT angiography, and RBC tag studies are various options that can pick the source of GIT bleed. Capsule endoscopy is a non-invasive tool to visualise mucosal surface of small bowel. However, this capsule is very large in size and it is difficult for a child to swallow. Diagnostic laparoscopy or exploratory laparotomy is one of the options in cases where the imaging studies and endoscopic evaluation fail to fetch diagnosis. Source of bleeding like Meckel’s diverticulum, duplication cyst, and vascular malformations etc. can be found. If no anatomic lesions are identified, one can perform peroperative small bowel enteroscopy to inspect small bowel mucosa.[1-5]

Blind loop syndrome is a well known problem and it usually presents with diarrhea, abdominal pain and fever.[4,5] It can lead to malabsorption, steatorrhea and deficiency of fat soluble vitamins.[5] However, presence of ulcers in the blind loops along with anastomotic ulcerations is extremely uncommon.[1-3] In our patient all the investigations could not direct us for the diagnosis however, exploration revealed the source of bleeding. To conclude, blind loops may grow to a significant length and may become symptomatic as happened in the index case.

## Footnotes

**Source of Support:** Nil

**Conflict of Interest:** None declared

